# Association between Selected Screening Tests and Knee Alignment in Single-Leg Tasks among Young Football Players

**DOI:** 10.3390/ijerph19116719

**Published:** 2022-05-31

**Authors:** Bartosz Wilczyński, Łukasz Radzimiński, Agnieszka Sobierajska-Rek, Katarzyna Zorena

**Affiliations:** 1 Department of Immunobiology and Environment Microbiology, Medical University of Gdansk, 80-219 Gdansk, Poland; katarzyna.zorena@gumed.edu.pl; 2 Department of Physiology and Biochemistry, Gdansk University of Physical Education and Sport, 80-336 Gdansk, Poland; lukaszradziminski@wp.pl; 3 Department of Rehabilitation Medicine, Faculty of Health Sciences with Institute of Maritime and Tropical Medicine, Medical University of Gdansk, 80-219 Gdansk, Poland; agnieszka.sobierajska-rek@gumed.edu.pl

**Keywords:** injury prevention, injury risk reduction, sports performance, dynamic knee valgus, knee kinetic

## Abstract

This study aimed to examine the relationship between knee valgus in the frontal plane projection angle (FPPA) during single-leg squat (SLS), single-leg landing (SLL), and other selected clinical tests in young athletes. Forty-three young healthy elite football players (age: 13.2 (1.7) years) that were regularly training in a local sports club participated in the study. The FPPA was assessed using 2D video analysis. The screening tests included the passive single-leg raise (PSLR), hip external and internal rotation (hip ER and IR), sit and reach test, weight-bearing lunge test (WBLT), modified star excursion balance test (mSEBT), countermovement jump (CMJ), single-leg hop for distance (SLHD), and age peak height velocity (APHV). There was a significant positive relationship between the knee valgus angles in the SLS test and the sit and reach test (r = 0.34) and a negative relationship with the hip ER ROM (r = −0.34) (*p* < 0.05). The knee valgus angles in the SLL were negatively associated with the hip IR (r = −0.32) and ER ROM (r = −0.34) and positive associated with the WBLT (r = 0.35) and sit and reach test (r = 0.33) (*p* < 0.05). Linear regression analysis showed that the results of the hip ER ROM and sit and reach tests were independent predictors of the FPPA in the SLS test (r^2^ = 0.11, *p* = 0.03 and r^2^ = 0.12, *p* = 0.02, respectively). The conducted study showed that individuals with more hip range of motion, more spine flexion extensibility, and less ankle dorsiflexion ROM may be more likely to experience high degrees of knee valgus in FPPA.

## 1. Introduction

Managing the development of young athletes is a challenging task due to the sports injuries that occur over the course of a career. Football players aged 13–19 years sustain 1 to 5 injuries per 1000 h of training and 15 to 20 injuries per 1000 h of match play. The vast majority of injuries are located in the lower extremities (60–90%) [1]. The injury rate increases in the range of 9 to 15 years with marked growth at age 13 among male footballers [1,2]. The occurrence of a serious injury throughout a career can turn away both young soccer players and parents from continuing to play the sport. Given that playing football promotes healthy lifestyles and has significant effects on decreasing risk factors for health diseases, injuries can steer players away from these benefits. Trying to reduce the occurrence of injuries in the future requires the use of appropriate prevention programs. To protect players, it is necessary to verify the factors corresponding to the risk of injury that can potentially be enhanced or minimized. Despite known and applied injury prevention programs (i.e., FIFA 11+ and the Prevention Exercise Program) [3,4], serious injuries, such as anterior cruciate ligament rupture, still occur in youth sports [3].

Furthermore, studies showed an increase in anterior cruciate ligament (ACL) injuries among adolescents in recent years [5,6,7]. Beck et al. found that the overall rate of ACL tears has increased annually by 2.3% over the past 20 years. Moreover, players aged 15–16 showed the greatest increase in ACL injuries [6].

Among adult male football players, the ACL injury rate showed no decreasing trend for several years. Furthermore, only 65% of players returned to their highest sporting level 3 years after ACL reconstruction, indicating a decline in sporting performance over a longer period [8]. Therefore, there is a need for new approaches to modify targeted prevention programs. Preventive training should focus on specific modifiable injury risk factors, such as decreased neuromuscular control, which manifests as dynamic knee valgus (DKV), among other signs [8]. DKV consists of concomitant movements of hip adduction and internal rotation, knee adduction, tibial external rotation, and ankle inversion with foot pronation. A DKV pattern is considered a risk factor for ACL injury and the occurrence of patellofemoral pain (PFP) [9,10,11,12]. Recent studies showed that some athletes who sustain an ACL injury land in a dynamic knee valgus position at the time of injury [13,14]. Moreover, young athletes who had increased knee valgus angles during a single-leg squat (SLS) are 2.7 times more likely to suffer from lower extremity injuries [15]. The gold standard for evaluating kinematic movement patterns of the knee is 3D analysis methods. However, one of the most popular and commonly used, low-cost, and simple evaluation methods is 2D analysis. Research showed high reliability and good agreement in terms of the mean difference between 2D and 3D analysis of the FPPA [16,17,18,19]. The FPPA of the knee is obtained via an angular measurement of the thigh and lower leg in the frontal plane in video analysis [19]. Therefore, in the current study, knee valgus evaluation was performed using the FPPA method. The knee valgus movement pattern can be observed in tasks such as cutting, landing, and squatting with one or two legs. However, due to the research evidence showing that ACL injuries mainly occur during single-leg tasks, we used single-leg squat (SLS) and single-leg landing (SLL) tests in our study [13,14].

To enhance lower extremity injury prevention programs among young athletes, it is important to examine what biomechanical variables may be related to knee valgus. One of these variables may be a joint range of motion. There exists a long list of joint range of motion tests performed in many sports clubs. Popular range of motion (ROM) tests with published inter-tester and test–retest reliability data include passive hip internal rotation (IR) and external rotation (ER), the weight-bearing lunge test (WBLT), passive straight leg raise (PSLR), and the sit and reach test [20]. Passive hip internal rotation range of motion (hip IR ROM) was described as a predictor of the knee valgus angle (FPPA) [21]. In one study, it was shown that higher values of the frontal plane knee projection angle (FPKPA) during an SLS were predicted by the hip abductor isometric torque and passive hip IR ROM among athletes [21]. Researchers indicated that subjects with adequate hip stiffness may have better knee alignment in the FPPA, despite reduced hip abductor torque [21]. Furthermore, athletes exhibiting increased hip IR ROM may be predisposed to greater internal rotation during activity, thus possibly initiating dynamic knee valgus [22]. Furthermore, Hogg et al. showed that a larger hip IR and ER ROM (together in combination) predicted greater peak knee internal rotation moments (which is a component of DKV) in adult females in the SLL test. However, the same study found that a smaller hip IR ROM predicted greater knee adduction moments (also a component of DKV) [23].

Restriction of ankle dorsiflexion range of motion (ankle DF ROM) can also affect knee valgus, as shown in the meta-analysis by Lima et al. [24]. The researchers revealed a significant relationship between weight-bearing and non-weight-bearing positions. Restriction of the DF ROM may engage forces toward compensation for excessive foot pronation, internal tibia rotation, adduction, hip internal rotation, and pelvic drop, leading to a full DKV pattern [24,25,26].

The next biomechanical features to consider in the context of knee valgus are spine and hamstring muscle flexibility. Popular and widely used tests to assess these features are the passive straight leg raise (PSLR) and the sit and reach test (SR). Additionally, the sit and reach test also evaluates spinal muscle extensibility [20]. Both features may be related to knee valgus in the FPPA. It is likely that the greater posterior muscle band stiffness may predispose someone to the greater trunk and lower limb stability and knee alignment in single-leg tasks.

The star excursion balance test (SEBT) and Y-balance test (YBT) are reliable and widely used scientific tests that are used to assess dynamic balance among athletes. Furthermore, a decreased YBT score is associated with the occurrence of lower limb injuries in various populations [27,28,29]. Dynamic balance may be another variable that may influence knee valgus. It seems that such a relationship may exist due to the fact that in these tests (SLS, SLL, and SEBT), participants have to use a similar ability to maintain dynamic one-legged balance. There are results obtained by Boey et al., who found no correlation between dynamic balance range and knee valgus moments among female netballers [30]. However, there is a lack of data regarding such a relationship among young footballers and in DKV assessment in more than one test.

Clinical screening tests, such as counter-movement jump (CMJ) and single-leg hop for distance (SLHD), are commonly used to examine sports performance. Jump performance tests are used to assess muscle power by examining jump distance and jump height. Furthermore, the recent study by Wilczyński et al. showed significant moderate positive correlations between the SLHD test and dynamic balance scores in YBT among young rugby players [31]. This correlation may be explained by similar neuropsychological structures being responsible for maintaining posture and producing lower limb power [32] and a similar strategy for developing lower limb strength abilities (ankle, knee, and hip extensor contractions during the flexion phase) [33]. Since the SLS and SLL tests also require the maintenance of a stable posture and the use of lower limb muscle strength, we assumed that a correlation with knee valgus angles might also occur.

Furthermore, biological maturation may be considered a confounding factor for knee valgus assessment due to changes in the neuromuscular control system during a growth spurt [34]. In the study by Ellenberger et al. 2020 that investigated DKV, the score of the drop jump test was related to individual biological maturation (maturity offset and peak height velocity), which explained 10.9% of the variance in DKV in young alpine skiers. Other anthropometric variables, such as age, body mass, or body mass index, were not associated with knee valgus. These results are in contrast to common assumptions that neuromuscular control improves with age. The authors also emphasized the requirement to use more and less demanding dynamic tasks in assessing knee valgus. In the case of our study single-leg landing and single-leg squat tasks were used [34].

Knowing the potentially modifiable and non-modifiable factors that predispose an athlete to DKV can help to establish effective lower limb injury prevention programs. From the standpoint of designing training programs and rehabilitation after injuries, it is important to know whether there is an association between FPPA of the knee, biological maturation, range of motion, dynamic balance, and power of the lower extremities. Significant correlations between these variables could contribute to a better understanding of the nature of dynamic knee valgus in FPPA.

Therefore, the purpose of our study was to investigate the relationship between factors such as the joint range of motion, jump performance, dynamic balance, biological maturation, and knee valgus in the FPPA in single-leg tasks. Our hypothesis was that a reduced ankle DF ROM and hip ROM, as well as greater spine mobility and hamstring muscle extensibility, would be associated with greater knee valgus. Furthermore, we hypothesized that athletes with decreased dynamic balance and lower limb muscle power values would exhibit greater angles of knee valgus in the FPPA.

## 2. Materials and Methods

### 2.1. Participants

Forty-three young male elite footballers recruited from a football academy met the inclusion criteria of our study. The anthropometric characteristics of the participants are presented in Table 1. The inclusion criteria were being aged between 12 and 15 (U12–U15 age groups) and having academy football experience (minimum 3 years). Participants who had sustained lower limb injuries or physical limitations in the last 6 months were excluded. All participants and their parents/guardians were informed in writing and signed consent to participate (including video recording) prior to participating in the study.

### 2.2. Ethical Approval

This study was part of a research project (clinicaltrials.gov number: NCT04780880) and was approved by the Independent Bioethics Committee for Scientific Research in Gdańsk (approval number: NKBBN/680/2020), which conformed to the principles embodied in the Declaration of Helsinki.

### 2.3. Procedures

All testing procedures took place in the sports hall of the Rehabilitation and Training Center. The tests were carried out in the afternoon from 12:00 to 5:00 pm over six days during the off-season period in December 2020. Participants attended four football training sessions (1.5 h each) per week during the off-season period with no games. During the season, the number and duration of the sessions remained equal, with the addition of a match on the weekend. All participants were fully rested and had not attended a training session or match in the last 48 h. Before the start of each test, participants were familiarized with the procedure. According to the test methodology, each participant performed “warm-up trials” before the actual trials.

All the tests were performed under the control of experienced physiotherapists and sports performance specialists. The examiners had at least 6 years of experience in performing the following tests (clinical and measurement experience). In addition, all examiners received additional training in both theory and practice from the principal investigator prior to the start of the study. Examination of all tests was conducted using standardized procedures to minimize the tester variability. Information on the experience of playing football was collected by the author’s questionnaire.

#### 2.3.1. Anthropometric Characteristics

The basic parameters (weight, height, body mass index) of the participants were recorded from the body composition analyzer (InBody 270, InBody Co., Seoul, Korea). The biological age was calculated from the body height, sitting height (assessed by measuring tape, 0.5 cm), body mass, and chronological age (study date) according to the formula of Mirwald et al. [35]. This formula also calculates the individual maturity offset prediction, which is a point in time before and after age peak height velocity (APHV).

#### 2.3.2. Single-Leg Squat and Single-Leg Landing

The single-leg squat and single-leg landing tests were carried out in accordance with the methodology described in previous studies [17,36,37,38]. All participants watched the instructional video and obtained verbal information. The researchers demonstrated correct squat and landing performance from the lateral perspective so that they did not suggest the knee’s projection from the frontal plane. After the instructions, the participants were able to practice the test (approximately four times) until they were ready to start [39]. The kinesiotape markers were attached to the anatomical points at the upper anterior iliac spine, at the middle point of the patella, and at the point of the ankle between the lateral and medial bones [17,36]. Three attempts were made for the left and right lower limbs with a two-minute recovery between squats. The mean of the three trials from the SLS and SLL tests were analyzed.

##### SLS

Participants were asked to perform a squat on one leg to approximately 60 degrees of knee flexion. During the test, participants had to stand barefoot on a designated line, have their non-test knee bent (approximately 90 degrees), keep their arms crossed on their shoulders, and look straight ahead. Then, the researchers signaled the start of the test, which could last a maximum of 5 s [36]. The trial was considered incorrect when the participants lost balance or supported themselves with another limb [39].

##### SLL

The participants were asked to jump off a 28 cm box with one leg, dropping vertically and landing in a designated area. When they lost their balance and touched the opposite limb to the surface for approximately 3 s, the attempt was repeated [37,38]. Additionally, the evaluators advised that the participants should keep their hands on their hips and land by flexing the knee at least 30°.

#### 2.3.3. Two-Dimensional Video Assessment

Knee alignment angles were analyzed using the frontal plane projection angle (FPPA) method. The FPPA assessment methodology was described in our previous study [39]. Two digital video cameras (GoProHero 4, GoPro, Inc., San Mateo, CA, USA) were placed on tripods. One camera was positioned laterally and the other frontally at a distance of 2 m from the study participants. Kinovea (beta-version 0.8.26, Bordeaux, France), which is a freely available motion tracking software, was used to assess the FPPA [18,40]. Kinovea was described as a validated and reliable method for measuring the angles and distances of human movements [41]. Markers were taped to the previously mentioned anatomical areas of the participants’ lower limbs.

**Figure 1:**
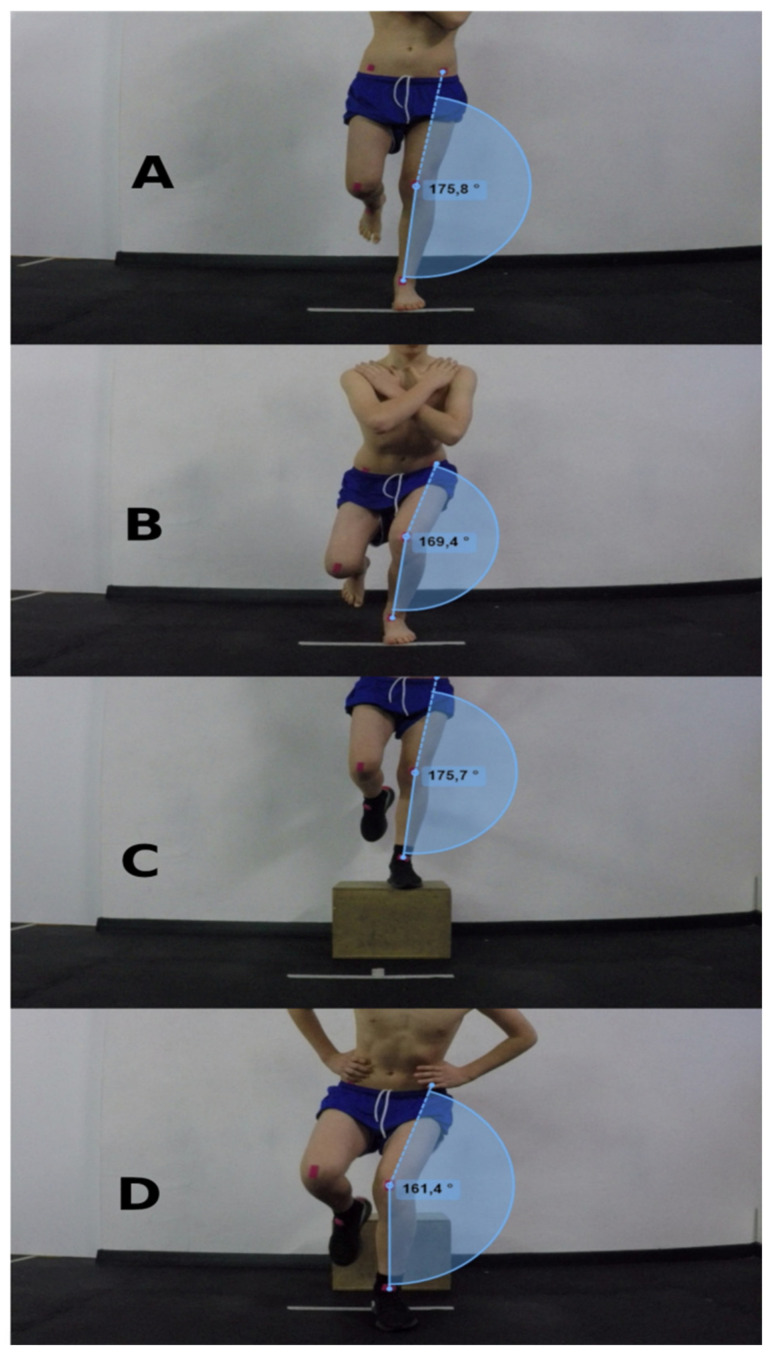
Example of the FPPA 2D assessment of knee valgus in the SLS and SLL tests. (**A**,**B**) SLS starting angle (176°) and end of the test (169°), and (**C**,**D**) SLL starting angle (176°) and end of the test (161°).

The researcher calculated the FPPA of the knee valgus angles during the video analysis, in which the angle was extracted using three points: the center of the patella, the upper anterior iliac spine, and the midpoint between the lateral and medial ankle bone (Figure 1). Analysis of the lateral projection (anatomical points: greater trochanter, lateral condyle of the tibia, lateral side of the ankle) allowed for assessment of whether the athlete had achieved the minimum knee flexion angle (at least 60 degrees). Previous studies showed good-to-excellent intra- and inter-session correlation of SLS and SLL scores with knee valgus (ICC = 0.72–0.91), with standard errors of measurement (SEM) of 1.97° for SLS values and 1.99° for SLL values [38].

#### 2.3.4. Range of Motion

Each passive range of motion assessment was done up to the end of the range. This may have been determined by the endpoint of range (tightness or firm resistance) reported by both the subject and/or the examiner. The rater measured the angles of the range of motion using a standard long-armed goniometer in increments of 1° or a tailor’s measure with an accuracy of 1 cm [42]. Three trials for each ROM test were performed and the mean of these scores was used for the statistical analysis. Participants did not perform a warm-up or any stretching exercises before the study.

##### WBLT

The weight-bearing lunge test (WBLT), which is based on the knee-to-wall principle, was used to assess the ankle dorsiflexion range of motion (ankle DF ROM). Participants performed 3 trials that involved touching the wall with the flexed knee in the lunge position from the most distant position of the foot. The heel of the subject’s leg could not lift from the floor and the foot needed to be on a designated line perpendicular to the wall. The maximum distance from the big toe to the wall was measured in centimeters. The mean of 3 trials was used for the analysis [43]. The WBLT demonstrated high inter-clinician (ICC = 0.80–0.99) and good intra-clinician reliability (ICC = 0.65–0.99) in previous studies [44].

##### PSLR

Hamstring muscle flexibility was evaluated with the passive single-leg raise (PSLR) test according to Kendall et al. [45]. While the participants were in the supine position, the rater assessed the angle of flexion of the hip joint with the knee extended relative to the torso. The maximum possible angle was measured with a goniometer and recorded in degrees [46]. It was shown that the inter-rater reliability of the PSLR test was at the level of ICC = 0.93 [20].

##### The Sit and Reach Test

The sit and reach test was used to assess the range of spine and hamstring mobility. The participants were instructed to sit upright against the wall and stretch their arms forward as far as possible and keep their hands on the chair for 1 s. One of the examiners checked whether the subject did not bend their knees and whether the heels remained in the starting position. The distance from the tip of the middle toe to the toe line was recorded and taken for analysis. If the subject touched the wall with the fingers, it meant 0 cm on the measurement scale, while larger values meant better test results. The test showed high inter-rater reliability at the level of ICC = 0.97 [20].

##### Passive Hip ER and IR ROM

Passive external rotation and internal hip rotation (hip ER and hip IR ROM) were measured with a handheld goniometer. The participants, lying supine on the plinth with the hip and knee flexed to 90 degrees, and the examiner, while stabilizing the limb, performed IR or ER until the end of the range of motion. The second tester assessed the rotation range in increments of 1.0 degrees [42].

#### 2.3.5. Dynamic Balance

The modified star excursion balance test (mSEBT) was used to assess dynamic balance. The participants were advised to stand in the center of the star formed by the intersecting four lines. Then the participants had to reach with their contralateral leg as far as they could along one of the lines in one of the three directions (anterior, posteromedial, posterolateral). The participants made 3 attempts in each direction for the right and left lower limbs. In order for the trial to be passed correctly, the participants had to keep their hands on their hips throughout the entire movement and maintain single-leg stability. The distance achieved by the subject (from the distal part of the big toe to the farthest point on the line) was measured in centimeters. The result was normalized to the length of the lower limbs and presented as a percentage. Lower limb length was measured from the anterior superior iliac spine to the lateral malleolus. The composite (COM) reach distance was calculated as the sum of three directions divided by three times the length of the lower limbs and then multiplied by 100. Previous studies demonstrated high interrater reliability in the assessment of the mSEBT reach distances [47,48].

#### 2.3.6. Jump Performance

##### CMJ

The countermovement jump (CMJ) test was used to assess the explosive strength of the lower limbs through the evaluation height of the vertical jump. The participants were instructed to stand on a contact mat (Fusion Sport Smart Jump mat, Fusion Sport, 2 Henley ST, Coopers Plains, QLD, 4108, Australia) with their hands on their hips before and during the jump [49]. The position to the jump (squat depth) was self-selected as in previous studies [50]. The test was passed when the participants straightened their knees during the flight and during the initial landing contact phase. There was a 2 min break between each jump [31]. Out of the three jump trials, the best trial was selected for the data analysis. The CMJ test was assessed as a reliable and valid flight-time method for assessing vertical jump height [51].

##### SLHD

The single-leg hop for distance (SLHD) is a test that is used to assess lower limb power using a horizontal single-leg hop. Participants were asked to stand with one leg on a line while keeping their hands on their hips. Immediately after the tester’s signal, the participants jumped as far as they could while landing on the same leg. The task was completed correctly when the players kept their balance without supporting any free limb for 2 s. The participants performed 3 jumps with a 30 s break between each jump. The tester assessed the distance of the jump using a tape measure and recorded it to the nearest 1 cm [31]. The average of the 3 trials was analyzed. The SLHD test demonstrated excellent test–retest reliability in a recent study [52].

### 2.4. Statistical Analysis

All variables were tested for a normal distribution using the Shapiro–Wilk test. Not all data were normally distributed, thus some of them were described as a median and interquartile range. Moreover, for this reason, both the Spearman rank test and the Pearson correlation coefficients were used for the correlation analysis. To determine the strength, the correlation coefficient was used (r): strong (0.50 ≤ r ≤ 1.0), moderate (0.3 ≤ r < 0.5), or weak relationship (r < 0.3) [53]. For factors that showed a significant Pearson’s correlation, the linear regression model was used to estimate the impact of the results. An independent samples Wilcoxon signed-rank test was used for the nonparametric data to analyze the differences in knee valgus in the FPPA between the SLS and SLL tests. All data analyses were processed with the Statistica software (Statistica 12). The significance level was set a priori at *p* < 0.05.

## 3. Results

### 3.1. Physical Characteristics

The characteristics of the physical performance and range of motion are shown in Table 2. Data from the left lower limb were used for the statistical analyses. The knee valgus angles (FPPA) were statistically significantly greater (*p* < 0.001) in the SLS test (16.4 ± 8.6) than in the SLL test (6.6, IQR 6.1).

### 3.2. Correlation between Variables and Knee Valgus Angle

**Figure 2:**
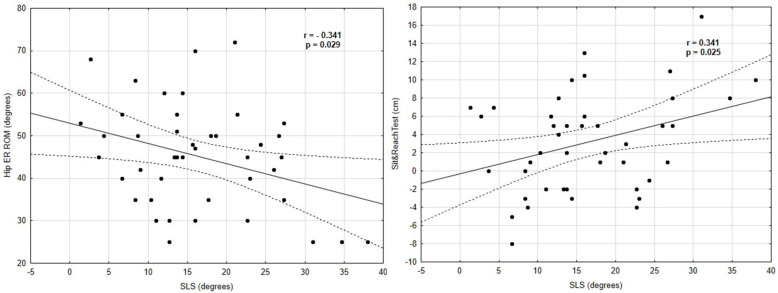
Correlations between the SLS and the hip ER ROM and sit and reach test results.

**Figure 3:**
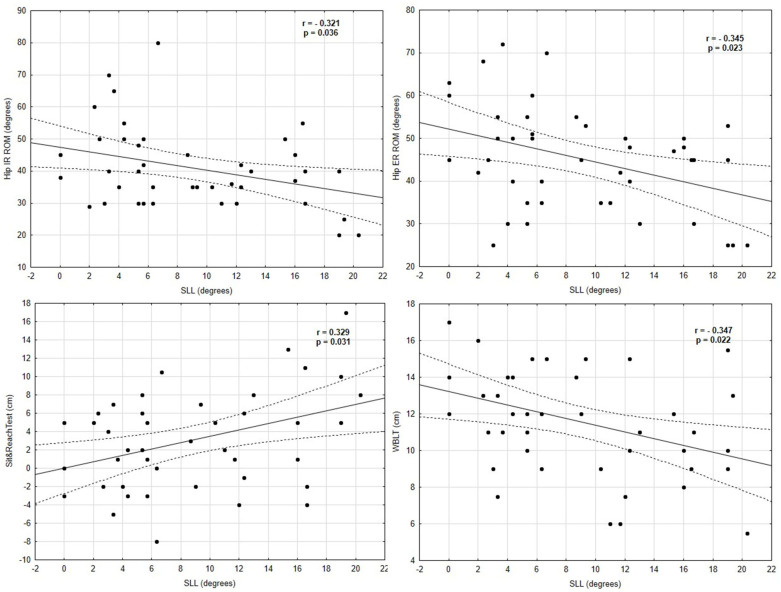
Correlations between the SLL and the hip ER ROM, hip IR ROM, sit and reach test, and WBLT results.

There were moderate negative correlations between the hip IR (r= − 0.32, *p* = 0.036) and hip ER ROM (r = − 0.34, *p* = 0.023) (where knee valgus increased as the hip IR and ER decreased), left WBLT (r = 0.35, *p* = 0.022), and dynamic valgus angles in the SLL test. Positive moderate relationships were found between the sit and reach score and dynamic valgus angles in the SLS (r = 0.34, *p* = 0.025) and SLL tests (r = 0.33, *p* = 0.031). A moderate negative correlation was found between the hip ER ROM (r = − 0.34, *p* = 0.029) and knee valgus angles in the SLS test (where knee valgus increased as the hip ER decreased). None of the dynamic balance scores, jump performance, or anthropometric variables (age, maturity offset, age peak height velocity) were associated with the dynamic knee valgus in both the SLS and SLL tests. All of the Pearson and Spearman correlations are presented in Table 3 and Table 4. Significant correlations between the variables and the SLS and SLL tests are shown in Figure 2 and Figure 3.

### 3.3. Predictor Variables for Knee Valgus Angles

Linear regression analysis was performed for parametric data that showed a statistically significant correlation with the results of knee valgus. The results of the hip ER ROM and sit and reach tests were independent predictors of the knee valgus angles in the SLS test (r^2^ = 0.11, *p* = 0.03 and r^2^ = 0.12, *p* = 0.02, respectively) (Table 5).

## 4. Discussion

Examining the specific factors associated with knee valgus in the FPPA may provide useful information for designing lower limb injury prevention programs. Therefore, the aim of our study was to investigate whether modifiable variables, namely, the joint range of motion and jump performance, and dynamic balance were associated with knee valgus in the SLS and SLL tests in young soccer players. The results of our study showed that there was an association of the FPPA in the SLL test with the hip ER and IR ROM and ankle DF ROM. For the FPPA in the SLS test, there was an association between the sit and reach score and hip ER ROM.

The FPPA in the SLL test showed a moderate negative correlation with the hip IR and ER ROM and ankle DF ROM, as well as the FPPA in the SLS with the hip ER ROM. The results of the association of the hip IR ROM with knee valgus were consistent with the results of Biencourt et al. [21] and Nakagawa et al. [26]. Furthermore, in a study by Flores-León et al., it was shown that the hip joint is associated with and may contribute to the dynamic knee valgus index in female football players [54]. Our data support the hypothesis that men exhibiting greater hip stiffness may have a relationship with higher angular knee valgus values in the FPPA. This may be due to the fact that limited mobility of the hip influences the occurrence of compensation at the knee joint, which manifests itself in greater dynamic knee valgus [10]. The effect of reduced ankle DF ROM on knee valgus has been demonstrated in previous studies [24,25,26,55]. In the study, Wyndow et al. showed that a lower ankle DF ROM was associated with a greater peak FPPA (greater valgus) on the SLS test in 30 healthy people [25]. The authors used the weight-bearing lunge test (WBLT), which measures the maximum distance between the longest toe of the foot and the wall that allows the knee to touch the wall without taking the heel off the ground, to assess the DF ROM [25]. Another study by Mauntel et al. showed that a group of active adults showing medial knee displacement on the SLS test had a lower passive ankle DF ROM than a control group [56]. In the study, Nakagawa et al. explained this phenomenon as a compensation strategy that forces pronation of the foot and, when foot the range is restricted, forces tibial movement in the transverse and frontal planes [26]. The results of our study were similar, but only for valgus in the SLL test. The lack of association of the ankle DF ROM with valgus in the SLS test was probably due to increased stability of the lower limb (particularly the ankle joint) compared with the SLL test; therefore, the compensation phenomenon may not have occurred.

Furthermore, our study found a positive moderate relationship between the sit and reach score and knee valgus angles in the SLS and SLL tests. Our participants in the sit and reach test showed relatively low scores (3.1 ± 5.4 cm). This may suggest that greater mobility of the spine and hamstring muscles may be beneficial for maintaining knee alignment during single-leg tasks. On the other hand, the range of hamstring muscle length in the PSLR test was not associated with knee valgus in both the SLS and SLL tests. Given that the sit and reach test examines spinal mobility in the direction of flexion and that the spine works inextricably with the hip joint, an explanation for the result could be that both the SLS and SLL are tests that require adequate flexion at the hip. Therefore, when subjects have limited trunk flexion (spinal mobility) they compensate for this movement at the lower segments of the kinematic chain, thus exhibiting greater knee valgus angles.

In addition, in the study of Miyamoto et al., the PSLR and sit and reach test showed only a weak-to-moderate correlation [57]. Therefore, we suspect that greater spinal mobility may have been the factor causing the association with the knee FPPA. When considering statistically significant correlation results for parametric data, linear regression analysis was performed. The analysis showed that the hip ER ROM and sit and reach test were the only predictors, explaining 12% and 11% of the knee valgus variability in the SLS test, respectively.

We also hypothesized that the reduced dynamic balance and decreased lower limb power in jump tests would correlate with higher knee FPPA values among participants. However, our study showed no significant statistical relationship between all the mSEBT directions, CMJ, SLHD, and knee valgus (in the FPPA) in the SLS and SLS tests. The lack of significant correlation between the mSEBT and DKV scores was similar to the results found by Boey et al. in female netballers [30]. The researchers found no correlation between valgus moments and the YBT results obtained by adult female netballers [30]. This result may explain the fact that both the single-leg landing and single-leg squat tests are tasks that require movement mainly in the sagittal plane and require less postural stability than the mSEBT. There was also no correlation between lower limb power in CMJ and SLHD and the DKV. This may have been due to the fact that the control of the knee alignment in the SLS and SLL tests requires more activity and muscular strength, e.g., of gluteal muscles [9], rather than strictly explosive strength of lower limb muscles. To our knowledge, there are no studies that have assessed the relationship between lower limb strength and knee valgus in single-leg tasks.

A recent study by Ellenberger et al. showed that biological maturation (maturity offset) was associated with and explained 10.9% of the variance in dynamic knee valgus during the drop jump test in young alpine skiers [34]. Moreover, Sonesson et al. found that youth football players showed reduced neuromuscular control compared with adult players, which can lead to greater DKV angles [58].

Our results are in contrast with the aforementioned results; there was no significant statistical association between the maturity offset, APHV, and knee valgus. The lack of association of knee valgus angles with age in our trial was similar to the results of Ellenberger et al. Thus, rejecting assumptions that increasing maturity (larger or smaller maturity offset) may be associated with increased neuromuscular control and maintenance of knee alignment.

It is also worth noting that the widely used 2D analysis offers many possibilities for individual assessment. Researchers are increasingly using it in studies on lower limb kinematics in different populations [59].

We confirmed the hypothesis that assumed a significant difference between valgus values in the SLS and SLL tests. Thus, we confirmed that high- and low-difficulty movement tasks should be used when testing knee valgus [34]. This result also explained why some of the same variables showed an association for only one test, i.e., either SLS or SLL.

## 5. Study Limitations

The present study had some limitations that we would like to address. Namely, in our study, we used the 2D analysis method of knee kinematic assessment. It is known that the gold standard for evaluating kinematic movement patterns of the knee is 3D analysis methods. However, one of the most popular and commonly used, low-cost, and simple evaluation methods is 2D analysis. The studies showed reliability and good agreement in terms of the mean difference between 2D and 3D analysis in the FPPA [16,59].

Second, the research presented was based on a small group of athletes and the lack of athletes with a history of no knee injury as a control group. Further cohort studies are being conducted to verify our findings and to garner a deeper understanding

Moreover, we believe that future studies on a larger population sample and for multiple sports may confirm our results. Furthermore, the nature of the study only allowed the parameters to be assessed at a single time point; therefore, future studies should try to follow changes over a longer period with repeated measurements.

## 6. Conclusions

Knee valgus in the FPPA in the SLL test was negatively correlated with the hip IR and ER ROMs and ankle DF ROM, and was positively correlated with the sit and reach test. Moreover, dynamic knee valgus in the SLS test was negatively correlated with the hip ER ROM and positively correlated with the sit and reach test. The FPPA in the SLS test was predicted by the hip ER ROM and sit and reach test. Individuals with a greater hip range of motion, greater flexion of the spine, and less ankle DF range of motion may be more likely to have greater knee valgus in the FPPA in single-leg movement tasks. In addition, our results showed that the FPPAs were greater in the SLS test than in the SLL test; therefore, in our opinion, the SLS and SLL tests should not be used interchangeably.

## Figures and Tables

**Table 1:** Anthropometric characteristics and football experience of participants.

Characteristics (*n* = 43)	Scores
Age (years), median (IQR)	13.2 (1.7)
Height (cm), mean (SD)	164.3 (9.3)
Body mass (kg), median (IQR)	45.7 (13.6)
APHV, mean (SD)	14.0 (0.6)
Maturity offset (years), mean (SD)	−0.6 (1.0)
Football training experience (years), median (IQR)	6.0 (3.0)

APHV—age peak height velocity.

**Table 2:** Characteristics of the range of motion, jump performance, and dynamic knee valgus of the participants.

Variables (*n* = 43)	Scores
**Range of Motion**	
PSLR (degrees), median (IQR)	75.0 (17.0)
Hip IR (degrees), median (IQR)	40.0 (18.0)
Hip ER (degrees), mean (SD)	45.2 (12.2)
Sit and Reach Test (cm), mean (SD)	3.1 (5.4)
WBLT (cm) median (IQR)	12.0 (5.0)
**mSEBT—Dynamic Balance**	
ANT (LL%), median (IQR)	68.5 (16.8)
PL (LL%), mean (SD)	99.8 (13.2)
PM (LL%), median (IQR)	98.2 (10.2)
COM (LL%), median (IQR)	89.0 (12.6)
**Jump Performance**	
CMJ (cm), mean (SD)	30.7 (4.9)
SLHD (cm), median (IQR)	184.3 (26.6)
**FPPA—Dynamic Valgus Angles**	
SLS (degrees), mean (SD)	16.4 (8.6)
SLL (degrees), median (IQR)	6.66 (11.33)

PSLR—passive straight-leg raise, hip IR and ER—hip internal rotation and external rotation, WBLT—weight-bearing lunge test, mSEBT—modified star excursion balance test, ANT—anterior, PL—posterolateral, PM—posteromedial, COM—composite, CMJ—countermovement jump, SLHD—single-leg hop for distance, FPPA—frontal plane projection angle, SLS—single-leg squat, SLL—single-leg landing.

**Table 3:** Correlations between the range of motion, anthropometric characteristics, jump performance, and knee valgus in the FPPA. PSLR—passive single-leg raise, hip IR—hip internal rotation, hip ER—hip external rotation, WBLT—weight-bearing lunge test; * and bold—statistically significant (*p* < 0.05), *p*-values were calculated using the ^a^ Pearson’s correlations and ^b^ Spearman Rank test.

Knee Valgus Angles (FPPA)				
	Single-Leg Squat		Single-Leg Landing	
	Correlation Coefficient	*p*-Value	Correlation Coefficient	*p*-Value
**Range of Motion**				
PSLR	−0.264 ^b^	0.086	−0.157 ^b^	0.313
Hip IR ROM	−0.114 ^b^	0.467	−0.321 ^b^	0.036 *
Hip ER ROM	−0.341 ^a^	0.029 *	−0.345 ^b^	0.023 *
Sit and Reach test	0.341 ^a^	0.025 *	0.329 ^b^	0.031 *
WBLT	0.009 ^b^	0.949	−0.347 ^b^	0.022 *
**Anthropometric characteristics**				
Age	−0.154 ^b^	0.323	−0.176 ^b^	0.323
Maturity offset	−0.141 ^a^	0.368	0.277 ^a^	0.860
APHV	−0.062 ^a^	0.694	−0.255 ^a^	0.098

PSLR—Passive straight-leg raise, Hip IR and ER—hip internal rotation and external rotation, WBLT—weight-bearing lunge test; * statistically significant (*p* < 0.05), *p*-values were calculated using ^a^ Pearson’s correlations and the ^b^ Spearman rank test.

**Table 4:** Correlations between the mSEBT, jump performance, functional movement screen, and dynamic knee valgus.

Knee Valgus Angles (FPPA)				
	Single-Leg Squat		Single-Leg Landing	
	Correlation Coefficient	*p*-Value	Correlation Coefficient	*p*-Value
**SEBT—Dynamic Balance**				
ANT	−0.066 ^b^	0.674	−0.149 ^b^	0.334
PL	0.076 ^a^	0.627	−0.085 ^b^	0.586
PM	−0.046 ^b^	0.768	−0.275 ^b^	0.074 *
COM left	−0.031 ^b^	0.841	−0.188 ^b^	0.226
COM right	−0.035 ^b^	0.822	−0.132 ^b^	0.397
**Jump Performance**				
CMJ	−0.232 ^a^	0.147	−0.115 ^b^	0.463
SLHD	−0.200 ^b^	0.198	−0.106 ^b^	0.499

mSEBT—modified star excursion balance test, ANT—anterior, PL—posterolateral, PM—posteromedial, COM—composite, CMJ—countermovement jump, SLHD—single-leg hop for distance, FPPA—frontal plane projection angle; * statistically significant (*p* < 0.05), *p*-values were calculated using ^a^ Pearson’s correlations and the ^b^ Spearman rank test.

**Table 5:** Linear regression model with Pearson’s correlation between the dominant leg SLHD and dominant leg YBT.

	r	r^2^	*p*-Value	Strength
**Single-Leg Squat**				
Hip ER ROM	0.336	0.113	0.027 *	Moderate
Sit and reach test	0.341	0.116	0.025 *	Moderate

* Statistically significant (*p* < 0.05).

## Data Availability

The data presented in this study are available on request from the corresponding author.
